# DNA based diagnostic for the quantification of sugarcane root DNA in the field

**DOI:** 10.1038/s41598-018-34844-3

**Published:** 2018-11-13

**Authors:** J. S. Pierre, D. Giblot-Ducray, A. C. McKay, D. M. Hartley, J. M. Perroux, A. L. Rae

**Affiliations:** 1 CSIRO Agriculture and Food, 306 Carmody Road, St Lucia, QLD 4067 Australia; 2 Molecular Diagnostics Centre, South Australian Research and Development Institute, 2b Hartley Grove, Urrbrae, SA 5064 Australia; 3 CSIRO National Research Collections Australia, Canberra, ACT 2601 Australia

## Abstract

Plant root systems play many key roles including nutrient and water uptake, interface with soil microorganisms and resistance to lodging. As for other crops, large and systematic studies of sugarcane root systems have always been hampered by the opaque and solid nature of the soil. In recent years, methods for efficient extraction of DNA from soil and for species-specific DNA amplification have been developed. Such tools could have potential to greatly improve root phenotyping and health diagnostic capability in sugarcane. In this paper, we present a fast, specific and efficient method for the quantification of sugarcane live root cells in soil samples. Previous studies were typically based on mass and length, so we established a calibration to convert root DNA quantity to live root mass. This diagnostic was validated on field samples and used to investigate the fate of the root system after harvest prior to regrowth of the ratoon crop. Two weeks after harvest, the sugarcane roots from the previous crop were still viable. This raises the question of the role that the root system of the harvested crop plays in the performance of the next crop and demonstrates how this test can be used to answer research questions.

## Introduction

Plant root systems play a key role in determining nutrient and water uptake and are also the interface between soil microorganisms and plants. In addition to these roles, the sugarcane root system plays an important part in anchorage of the plant to provide resistance to lodging during the cropping season and at harvesting. Because of the perennial nature of the crop, sugarcane roots developed by one crop may be important for the next ratoon, at least at the start of the next crop since the fate of sugarcane root systems between crop cycles is unclear^1,2^.

As for any other crop, the opaque and solid nature of the soil has always been a major limitation for the study of sugarcane root systems. The size of the plant as well as the length of the cropping season also limit the size of experiments in controlled environments. Consequently, knowledge of sugarcane roots is sparse.

Field phenotyping of root systems presents many challenges. Shallow root cores are often not representative of entire root systems^3^. Samples collected either with a large diameter (50 cm) surface corer^4^ or a 2 m auger^5^ need to be processed in a way that retains root material and structural information. Fine roots are easily lost and underrepresented^3,6^. These fine roots make up around 90% of the root system total length and are the elements responsible for water and nutrient absorption^6,7^. Measurement of root system basic descriptors such as root length, volume and diameter is also a slow process (up to 3 hours per plant) that does not give any information regarding whether a root is dead or alive. Due to these limitations, most of the analyses done so far on sugarcane have been restricted to a limited number of varieties and replicates, using small soil samples to monitor root distribution in the ground^1,8–11^. Many critical questions about sugarcane root systems, including inter-varietal variation and dynamic changes in the field remain unanswered. Hence plant breeders lack the knowledge and tools to select for beneficial root traits and growers lack a root diagnostic tool that will assist them with monitoring their crop’s below ground development and health.

With the harnessing of molecular biology for diagnostics, methods for efficient extraction of DNA from soil and for species-specific DNA amplification have been developed for routine use^12^. These tools have been mainly used for research and for soil-borne pathogen detection^12^ but have also been applied to quantify DNA from seed and roots in soil^13^ and more recently to study varietal differences in root growth in glasshouse and field trials^14–19^. The techniques have the advantage of reporting DNA concentration in soil associated with living roots and are compatible with the high-throughput system developed by commercial services^12^. Such tools could be adapted for sugarcane and have the potential to greatly improve root phenotyping and health diagnostic capability.

In this paper we present the results of the development of a TaqMan assay for the quantification of sugarcane root DNA in soil, its calibration to report the assay results as root mass, and its validation on field samples.

## Result

### TaqMan assay specificity test

The assay detected sugarcane DNA efficiently as evidenced by the cycle threshold (Ct) values ranging from 16.6 to 19.2 for 22.8–26.4 ηg sugarcane DNA per reaction (Table 1). In contrast, the level of amplification with 20 ηg of weed DNA matrix per PCR was very low, with Ct values ranging from 33.5 to 37.8 Ct. This represents a sensitivity difference of 100,000 to 1,000,000.

**Table 1:** TaqMan assay sensitivity and specificity assessment using four sugarcane varieties and eight common weeds of sugarcane cultivation. Cycle threshold (Ct) mean values were calculated based on 3 replicates.

Sugarcane variety	DNA quantity (ng)	Ct mean
Q208	26.4	16.6
MQ239	23.6	19.2
KQ228	23.2	17.9
Q242	22.8	17
**Weed**		
*Brachiaria subquadripara*	20	33.5
*Chloris gayana*	20	35.7
*Cynodon dactylon*	20	35.7
*Digitaria ciliaris*	20	37.5
*Echinochloa colona*	20	36.5
*Eleusine indica*	20	34.8
*Panicum maximum* cv *guinea*	20	35.1
*Panicum maximum* cv *nami*	20	37.8

### ITS copy number

In the 31 sugarcane varieties tested, ITS copy number per ρg of DNA ranged from 476 ± 7 (Q249) to 1333 ± 44 (Q208) with a median ITS copy number of 681 (Table 2). The relative standard deviation of the mean was on average below 1.8%. ITS copy number was significantly different between varieties (p < 0.001). The variety with the lowest ITS copy number (Q249) was used as a reference to quantify differences between varieties; the ITS copy number was 1.05 to 2.80 times higher in other varieties compared to Q249. The median scaling factor was 1.43, with only three varieties (Q124, Q113 and Q208) out of 31 having a scaling factor above 2.

**Table 2:** Genetic variation in ITS copy number amongst 31 sugarcane cultivars. Scaling factor represents the ITS copy number normalized for each variety by the ITS copy number of Q249.

Cultivar	Year first planted as seedling	Mean ITS copy number ± sd (copy ρg^−1^)	Scaling factor
Q249	2002	476 ± 7	1
Pindar	NA	500 ± 9	1.05
Co290	<1950	507 ± 5	1.07
Q96	1959	528 ± 5	1.11
Q117	1963	568 ± 9	1.19
MQ239	1993	584 ± 11	1.23
QC91-580	1991	591 ± 10	1.24
Q231	1995	617 ± 25	1.3
Q256	2002	618 ± 11	1.3
Q190	1986	624 ± 19	1.31
Q252	2000	638 ± 13	1.34
Q151	1981	652 ± 13	1.37
Q242	1997	660 ± 8	1.39
Q232	1994	662 ± 18	1.39
Q167	1977	674 ± 9	1.42
Q77	1940	681 ± 6	1.43
EMPIRE	<2003	689 ± 8	1.45
POJ2878	<1930	692 ± 6	1.45
Q200	1989	698 ± 17	1.47
KQ228	1998	702 ± 7	1.47
SRA1	2005	704 ± 10	1.48
Comus	<1942	743 ± 13	1.56
QN04-121	2004	748 ± 35	1.57
QBYCO5-20853	NA	753 ± 12	1.58
Q138	1975	794 ± 13	1.67
QN04-668	2004	798 ± 7	1.68
Q234	1988	818 ± 11	1.72
NCo310	NA	907 ± 17	1.91
Q124	1969	1119 ± 30	2.35
Q113	1967	1305 ± 23	2.74
Q208	1987	1333 ± 44	2.8

### Assay calibration

The aim of the assay calibration was to determine the coefficient of the linear relationship between root mass and the TaqMan assay results, which are expressed as DNA copy number, for several varieties.

**Figure 1:**
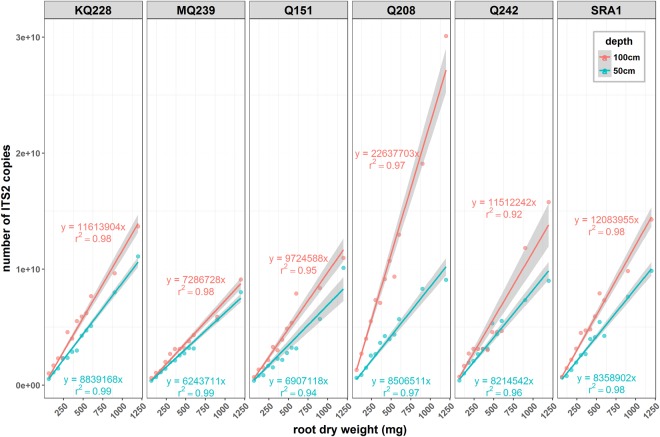
Relationship between the root dry weight and the quantity of target DNA extracted and measured from root samples from six sugarcane varieties (n = 3). Root samples were collected either from the upper part (0–50 cm from the crown; blue) or from the lower part (50–100 cm from the crown; red) of the root system. Calibration was done on root dry weight ranging from 60 mg to 600 mg with 60 mg increments as well as two additional points at 900 and 1200 mg. Linear regressions were fitted for each depth using a null intercept to obtain a calibration factor (slope). Grey zones represent the 95% confidence interval for prediction.

Assay calibration results are presented in Fig. 1. For all varieties and depths tested, the correlations between root dry weight and target DNA copy number all had a coefficient of determination (r^2^) above 0.95 except one (Q242 50–100 cm depth; r^2^ = 0.92). Based on these correlations, the assay efficiency was calculated to be on average 97%. Depth had a significant effect on the slopes of the linear regression (p < 0.001). The DNA copy number was higher for the lower part of the root system (50–100 cm) compared to the upper part (0–50 cm). There was also a variety effect (p < 0.001) with MQ239 and Q151 root systems apparently yielding fewer target DNA copies per mass unit of root. One out of 144 samples showed a slight PCR inhibition but results from all samples were used for the statistical analysis.

**Figure 2:**
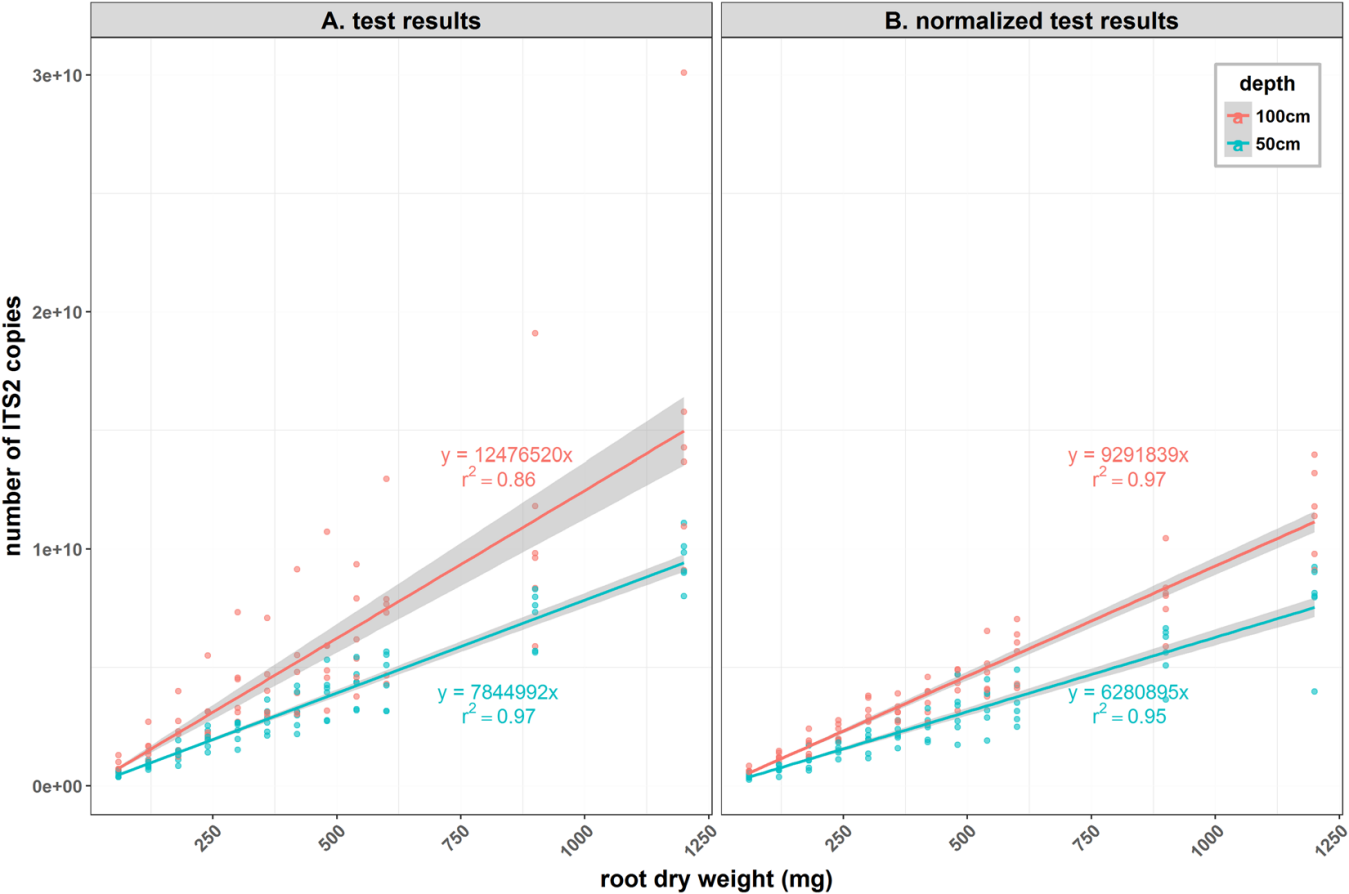
Linear relationship between root dry weight and the quantity of target DNA extracted and measured for all varieties combined for each depth, before (**A**) and after (**B**) normalization to take into account varietal differences in ITS copy number. Grey zones represent the 95% confidence interval for prediction.

When combining data of all varieties for each depth, the coefficient of determination of the linear regression was higher for the 0–50 cm samples (r^2^ = 0.97) compared to the 50–100 cm samples (r^2^ = 0.86) (Fig. 2A). After correction for varietal differences in ITS copy number, both regressions had a coefficient of determination above 0.95 (Fig. 2B).

### Root DNA decay over time

**Figure 3:**
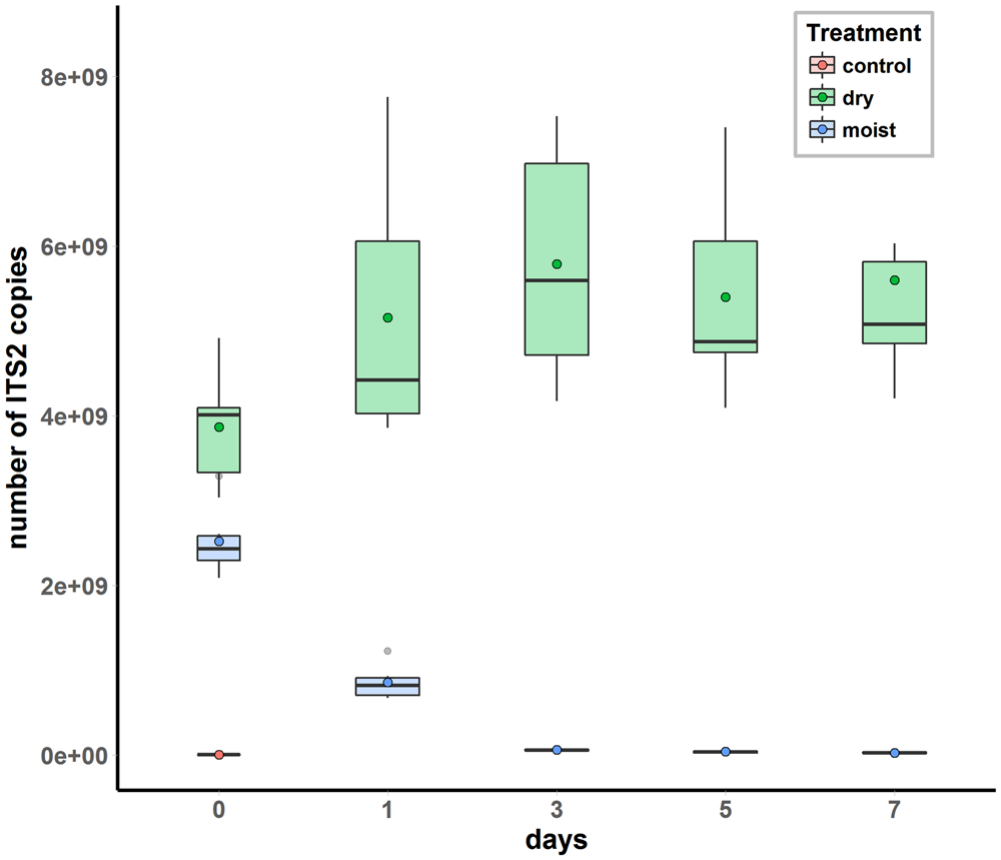
Root DNA decay over time in moist (blue) and dry (green) soil samples (n = 6 per boxplot) spiked with 200 mg of lyophilized sugarcane roots. Samples were incubated at 25 °C in a growth cabinet for 0, 1, 3, 5 and 7 days. After incubation, the samples were transferred to a dehydrating oven at 45 °C until analysis. Un-spiked moist soil samples (n = 2) were used as a negative control.

The ability of the assay to discriminate between live and dead roots was tested by measuring the decay of root DNA in moist and dry soil samples spiked with sugarcane root segments (Fig. 3). Root DNA quantity in the moist soil samples declined rapidly and significantly over time (p < 0.001) (Fig. 3). After 1 and 7 days the quantity of root DNA in the moist soil samples had declined by 66.6% ± 8.2 and 98.9% ± 0.5 respectively, compared to the DNA quantity at the zero time point (T_0_) in the moist samples. In contrast, the amount of root DNA in the dry soil samples was stable and did not significantly vary over the seven days (p = 0.131). There was also a significant difference (p = 0.002) at T_0_ between the dry and moist samples. DNA quantity measured in a control sample with no added roots was minimal, corresponding to 0.05% of the quantity of DNA at T0 in the moist sample. All samples passed the internal control quality check.

### Effect of nitrogen fertiliser on root systems

The DNA assay was used to assess soil core samples collected from a field trial that was designed to test the effect of different types of nitrogen input on sugarcane yield. The aims of this experiment were to (i) test the effect of the sampling method on root DNA estimation, and (ii) describe sugarcane root profiles under three nitrogen fertilization regimes.

DNA assay results were converted to root quantities using the Q208-50 cm linear regression equation presented in Fig. 1. Results from all samples were used for this analysis as all DNA passed the internal control quality check.

There was no significant difference in the calculation of root quantities (p = 0.169) (data not shown) if this conversion was done instead using the 50 cm linear regression equation obtained from the general calibration before (Fig. 2A) or after ITS copy number correction (Fig. 2B).

**Figure 4:**
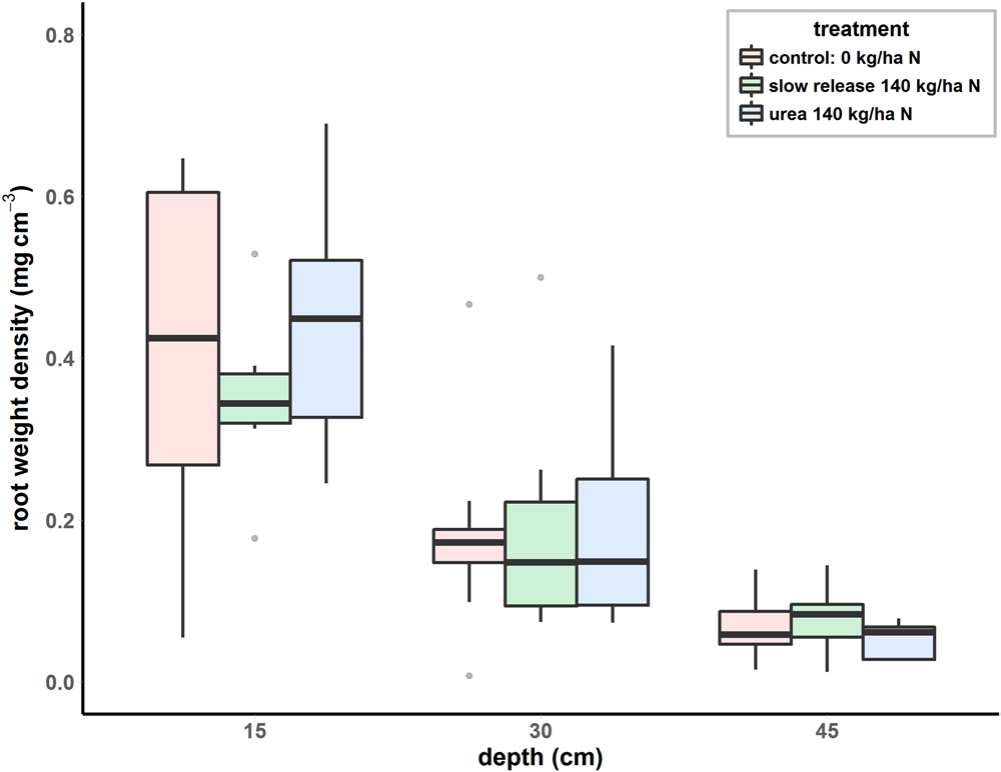
Root distribution in the soil profile expressed as root weight density for a ratoon crop (variety Q208) fertilized with N at 140 kg ha-1 as slow release fertilizer (green) or as urea (blue) or without any fertilizer added (red). Each sample (n = 9 per boxplot) is a composite of 12 soil cores (150 mm × Ø15 mm). Root weight density values were calculated using the Q208-50 cm calibration factor and an average soil bulk density of 1.5 g cm^−3^.

The amount of root detected in the composite samples ranged from 2.4 mg to 275.6 mg which corresponds to 0.008 and 1.007 mg of root per cm^3^ of soil respectively (Fig. 4). There was no treatment effect on root quantities (p = 0.724) and for a given set of conditions, the variability was large with a relative standard deviation of 57% on average.

There was a depth effect on root density (p < 0.001) with a sharp decrease down the soil profile for each of the three different treatments. On average, the root mass per volume of soil decreased for each depth increment from 0.43 g cm^−3^ to 0.19 g cm^−3^ and to 0.07 g cm^−3^ (Fig. 4). This corresponds to an average of 55% and 84% decrease in root quantity when comparing results from the 0–15 cm cores with results from the 15–30 cm and 30–45 cm soil cores respectively.

### Root viability after harvest

**Figure 5:**
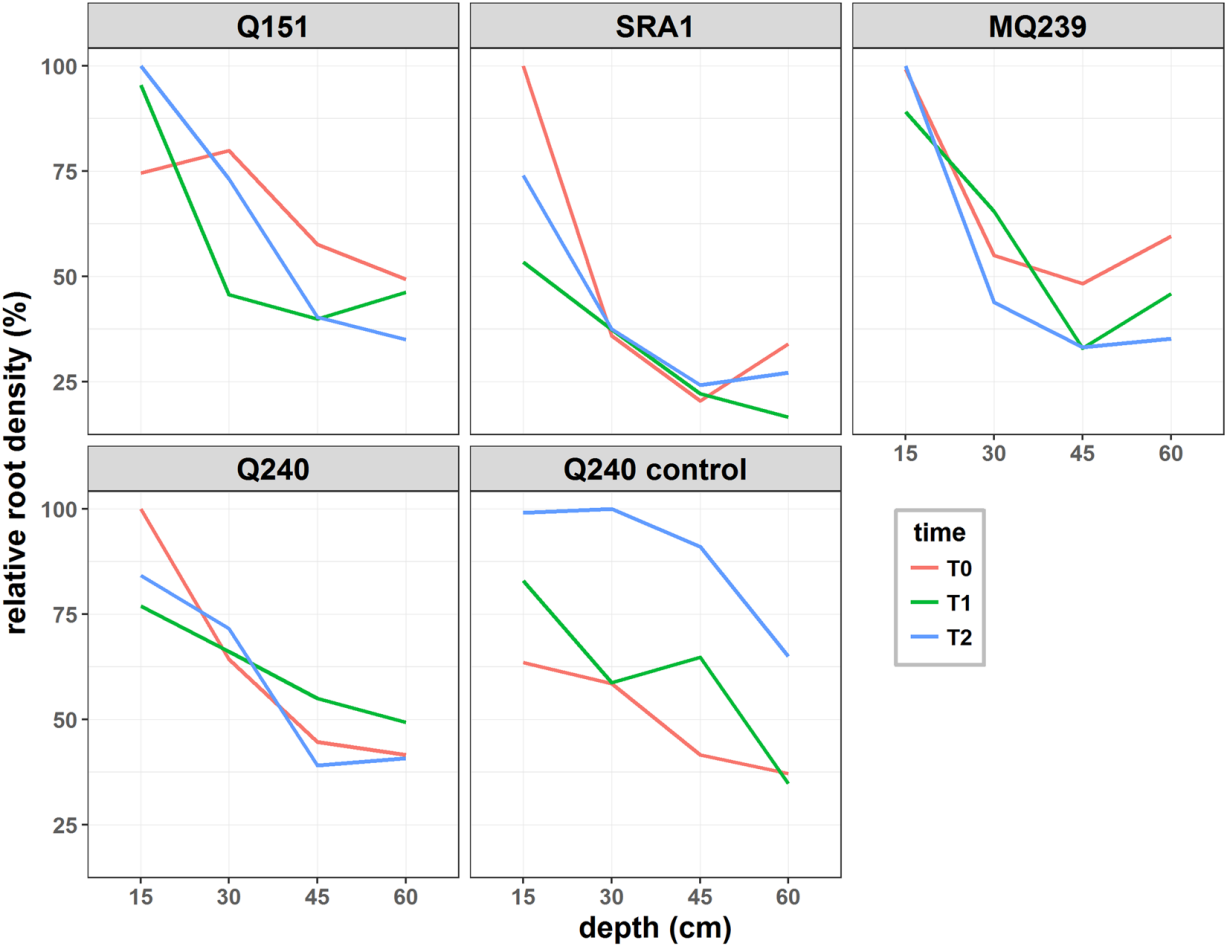
Root distribution in the soil profile at harvest (T0; red) and one week (T1; green) or two weeks (T2; blue) after harvest for four sugarcane varieties and an uncut control (Q240 control). Each sample is a composite of 8 soil cores (150 mm × Ø15 mm) collected on four different plants (2 cores per plant). The same plants were sampled for the entire course of the experiment. The relative percentage of root in each layer at each time points was calculated according to the maximum amount of root in any sample for a given variety.

The changes in sugarcane viable root mass in the soil was monitored over two weeks after harvest of the above-ground biomass (Fig. 5). The decline in root mass was positively and significantly correlated with the depth of the soil core sample (p < 0.001) whereas time did not have a significant effect (p = 0.442) on the quantity of viable sugarcane root in the soil over the two week period. When looking only at the uncut control, there was a significant increase in root mass between T0 and T2 (p = 0.024).

## Discussion

This study presents a novel method for the quantification of sugarcane root biomass in soil. The method was calibrated for a range of sugarcane varieties and validated with field samples. Results show that the method is robust, specific to sugarcane and can detect small amounts of live roots in soil samples, which will enable high throughput field phenotyping of sugarcane root systems.

### Varietal difference in ITS copy number is limited in sugarcane

Since the assay relies on the amplification of the ITS sequences in real time PCR, the ITS copy number directly influences the quantification results. Determination of the ITS copy number for each variety was an important step in the development of the assay to normalise results to account for potential large differences between varieties and enable multiple varieties comparison. In a previous study on wheat^16^, ITS copy number has been quantified by using a known single-copy gene as a reference. In polyploid sugarcane, in the absence of single-copy genes, we used ddPCR as a novel method to quantify ITS copy number. This technology allows for the absolute quantification of a DNA fragment of interest. When we quantified the ITS copy number for the 31 varieties, the relative standard deviation of the mean was below 1.8% on average. This demonstrated that, providing enough technical replicates, ddPCR gives reproducible results and could be used in the future to calibrate the assay for new varieties, as an alternative to the single copy gene method. Interestingly, the difference in ITS copy number amongst Australian bred varieties was similar to that between international bred varieties, which suggests that the assay can be used worldwide for sugarcane root phenotyping.

Overall, the extent of the ITS copy number variation, amongst the 31 varieties tested, was quite limited. The average copy number variation was 1.5-fold compared to the variety with the lowest copy number, and only three varieties exceeded a 2-fold difference. In comparison, the ITS copy number variation amongst 20 wheat cultivars ranged from 2 to 6 fold with an average fold increase of 3.9^16^. Therefore using a scaling factor to correct for ITS copy number when comparing multiple varieties of sugarcane may not be necessary, given the extent of the variability resulting from other factors in the experiment.

### Relationship between root biomass and test results

The calibration of the test to obtain the relationship between root biomass and DNA test results highlighted that for the same mass of root, the lower part of the root system was about 1.5 times more dense in DNA than the top part of the root system. This depth effect could be explained by changes to the root anatomy, particularly the increasing proportion of root cortical aerenchyma in ageing shoot roots^20^. As the roots age, cortical cells are progressively lysed until no cortical cells are left except for some cell wall bridges to maintain root structural integrity^21^. The only living cells at this stage come from the peripheral tissues and the endodermis^20^. This structure is characteristic of older root segments which primarily function in transport and not in uptake of water and nutrients^7^. In our experiment, the lower part of the root system, being richer in root apical segments with intact cortex, likely has a higher proportion of living cells and consequently higher DNA content, which would explain the observed depth effect on the calibration. The six varieties selected from this experiment have been selected on the basis of their contrasted root system morphology, especially in terms of average root diameter which relates to cortical cell number (Pierre *et al*., unpublished). Hence, the differences of DNA copies per mass unit of root observed in our experiments were likely due to varietal differences in root tissue density and the number of cortical cells per mass unit rather than being the result of differences in DNA extraction efficiency between varieties.

### Root DNA decay in the soil

To determine the ability of the test to discriminate live roots from dead roots, we quantified the decline in DNA content in dry and moist samples spiked with segments of lyophilized sugarcane roots. The results showed that DNA degradation in moist soil samples is an extremely fast process. After seven days, only 1% of DNA was still detectable in the moist soil samples, while during the same period, no DNA degradation occurred in the dry samples.

Previously, using the same technique^17^, it has been shown that there was an 80% decline in ryegrass DNA levels in soil over a 10-day period after plants had been defoliated or sprayed with glyphosate and that the DNA test was able to detect root mortality when a visual assessment could not. Similarly, an exponential decline in *Phalaris* DNA^15^ was observed over a 7-day period in soil spiked with *Phalaris* roots. This indicates that as long as soil moisture is controlled for, the DNA assay is able to discriminate between live and dead roots.

While the quantity of starting material was the same for the dry and moist samples, the DNA quantity at T_0_ was significantly different between the two treatments. At T_0_, when the experiment was set up, all the samples stayed at room temperature for approximately 3 h until all were prepared. Hence the T_0_ moist samples had incubated for approximately 3 h before being dehydrated at 45 °C. Since DNA degradation in moist soil is a rapid process, this would explain the difference in DNA quantity at T_0_. This observation reinforces the idea that in order to minimize DNA degradation, the samples (i) should be kept on ice as soon as they are collected; and (ii) need to be dehydrated in an oven with an exhaust air function to remove water rapidly^19^.

### Field sampling variability

With field samples (nitrogen experiment), the DNA assay proved to be extremely sensitive and detected small amounts of root in soil samples, with quantities as low as 0.007 mg of dry root per cm^3^ of soil. The results also described well the typical decrease in root mass from the surface down the soil profile in sugarcane^1,11^. The overall average root density estimated in the 7-months old ratoon crop used in the experiment was comparable to the one obtained by Otto *et al*.^1^ from samples collected on 15 month-old sugarcane crop. At this stage of the crop cycle, there was no difference in root density between the different fertilization treatments. This observation is consistent with the results from two experiments conducted in Brazil on planted and ratoon crops^22^, where no consistent positive effect of nitrogen was observed on root mass. Another explanation for the lack of apparent effect of nitrogen fertilizer on the root biomass could be the large intra-sample variability resulting from the sampling procedure. As mentioned by Steinemann (2016) an optimized field sampling protocol relies on the choice of sampling equipment, the core diameter and the number of cores to be taken. In the present study the sampling was done manually using a soil probe hammered down with a sledge hammer. The position of the core was as close as possible to the sugarcane stool but was somewhat variable due to large differences in plant size and stalk number which limited the access to the base of the plant.

The position effect was evident in the root turnover experiment where there was a significant increase in root mass for the uncut control plants at T2. While for all treatments and varieties the samples were collected close to the stool for the three time points, this was only possible at T2 for the control, due to the size of the stool and the number of stalks when the plants were still standing. This may explain the sudden increase in root mass and highlights the need to develop field sampling strategies that rely on automatic core sampling devices and consistent sampling positions.

### Sugarcane roots are still alive two weeks after harvest

Due to the perennial nature of sugarcane and its crop cycle, the fate of the roots after harvest is highly relevant to ratoon performance. Several studies on field grown sugarcane investigated root cycle^8^ and results from these are currently used to quantify root dieback after harvest in the APSIM sugarcane crop model^23^. In these studies, root viability was determined based on recovery of roots from soil and on the root physical appearance, principally colours. There are two pitfalls with these types of assessment. First, washing roots out of soil cores is a slow and inefficient process that leads to the partial loss of the fine roots^6^ which are the absorptive part of the root system. Secondly, determining root viability based on their colours is operator dependant and subjective. In a study on tree species, 12–28% of roots were mistaken for dead, based on their colour, while they were still metabolically active^24^. The use of the DNA test overcomes the first pitfall by avoiding root washing as well as the second one, by purely defining root viability in terms of DNA presence or absence.

In the root turnover experiment, there was no evidence of dieback for up to two weeks after harvest for the four varieties tested. This will need confirmation with a follow up experiment over a longer time course and with higher number of replicates to reduce the variability around the results. Our initial hypothesis was that after harvest the existing root system would die and be replaced quickly by new roots from developing tillers; results suggest that this may not be the case. While results need to be confirmed, this raises questions about the role of the former root system in the performance of the following crop as well as how the root system is maintained in the absence of a supply of photo-assimilate during re-sprouting. A recent study in longleaf pine^25^ demonstrated that, when girdling the stem to stop the flow of photo-assimilate toward the root system, fine roots could survive for over a year with the energy provided by the non-structural carbohydrate coming from the transport roots. In a mature sugarcane plant, the sucrose content ranges from 14 to 42% of the stalk dry weight^26^. When sugarcane is harvested, a fair proportion of the underground stool contains stalk bases rich in sucrose which could therefore fuel the root. This hypothesis will have to be tested.

In conclusion, we developed a rapid, reliable and sensitive method to quantify viable sugarcane root mass in soil. While further development is required on a sampling strategy to reduce the error associated with difference in sampling positions, this method can be readily used with the current calibration to reliably assess field samples. In the future, by combining our root phenotyping method with a similar one focusing on the quantification of soil borne disease, we would be able to assess root health. It will also be possible to develop a calibration based only on absorptive root length. By mapping absorptive root length density in the soil together with other soil parameters (*e.g*. water, nutrients, microbiota) this has the potential to unlock root phenotyping capability in sugarcane.

## Materials and Methods

### Development of the sugarcane-specific quantitative PCR primers and TaqMan probe

In order to design PCR primers for the cloning and sequencing of the Internal Transcribed Spacer (ITS) region of sugarcane varieties, 184 sequences of the ITS1-5.8S-ITS2 region from sugarcane and its relatives were retrieved (Table 3) from the NCBI database. A consensus sequence was obtained from the alignment of the 185 sequences performed in CLC Main Workbench7 (Qiagen, Hilden, Germany). Forward (5′TCGTGACCCTTAAACAAAACAGACCGC3′) and reverse (5′AGCGGCTATGCGCTACGGT 3′) PCR primers were designed based on the consensus sequence.

**Table 3:** Origin and number of ITS sequences retrieved from the NCBI nucleotide database from sugarcane and its relatives used to obtain a consensus sequence for the cloning of the ITS1-5.8S-ITS2 region in sugarcane.

Species	N° of sequence
Saccharum spontaneum	104
Saccharum sinense	9
Saccharum robustum	5
Saccharum officinarum	10
Saccharum longesetosum	2
Saccharum hybrid cultivar	22
Saccharum giganteum	2
Saccharum fulvum	3
Saccharum fallax	1
Saccharum brevibarbe var. contortum	1
Saccharum barberi	6
Saccharum baldwinii	2
Saccharum arundinaceum	17

Total DNA was isolated from freeze-dried leaves (Plant mini kit, Qiagen, Hilden, Germany) of 18 sugarcane varieties (KQ228, MQ239, Q138, Q183, Q186, Q190, Q200, Q203, Q208, Q219, Q226, Q231, Q232, Q237, Q238, Q240, Q241, Q242). The ITS1-5.8S-ITS2 amplified DNA fragment was cloned in pGEM-T easy vector which was then used to transform JM109 competent cells. Cells were then plated on LB/ampicillin/IPTG/X-gal medium in petri dishes. After 24 h at 37 °C, two positive colonies (white) were picked for each transformation and cultivated in liquid LB culture overnight. Plasmid DNA was purified using the QIAprep Spin Miniprep Kit (Qiagen) and the presence of the insert was tested using a restriction digest with *Not1*. Thirty-six positive plasmid samples were sent to a commercial service for Sanger sequencing. Sequencing results were used to obtain the consensus sequence of ITS1 and ITS2 (Supplementary S1).

qPCR primers and 6-FAM-labelled TaqMan Minor Groove Binding (MGB) probe (Table 4) were designed using the Primer Express 3.01 program (Thermo Fisher, Waltham, USA). Primers and probe were tested for homology against plant DNA sequences from the NCBI database using Blastn to check for any cross reactions of the assay with ITS sequences from any of the major weeds of sugarcane cultivation. qPCR reactions to test TaqMan assay were performed with an ABI ViiA7 instrument using the Qiagen QuantiTect probe PCR kit master mix according to the manufacturer’s instructions. Briefly, 10 μL reaction mixes contained 5 μL of 2x Qiagen QuantiTect Probe PCR kit Master Mix; 0.16 μL of water; 0.22 μL of 18 μM forward primer; 0.22 μL of 18 μM reverse primer; 0.4 μL of 5 μM probe and 4 μL of DNA template. Thermal cycling conditions were an initial step at 95 °C for 15 min followed by 40 cycles of melting step at 95 °C for 15 s and combined annealing and extension step at 60 °C for 1 min.

**Table 4:** TaqMan assay primer and probe sequences.

Assay	Name	Function	Sequence (5′ > 3′)	Length	Tm (°C)
ITS2-pb2	Fp_A5	forward primer	AAAAGACACTCCCAACCCAC	20	60
	Rp_A5	reverse primer	ACCGAGAACAACTGAGTGTC	20	60
	Pb_A2	MBG TaqMan probe	CCGGCGAATCGTGT	14	69

The specificity of the assay toward sugarcane was tested using eight grass weeds of worldwide significance that are common weeds in Australian sugarcane growing regions. (listed in Table 1). Weed DNA was extracted from freeze-dried leaf samples using a Qiagen DNeasy plant mini kit. Real-time PCR reactions were conducted, in triplicate, with a quantity of either 20 ηg of weed DNA or approximately 25 ηg of sugarcane DNA per well.

### Sugarcane ITS copy number quantification

In the absence of a single copy reference gene in sugarcane, ITS2 copy number was quantified using droplet digital PCR (ddPCR). Thirty-one sugarcane varieties (Table 2) were selected to represent varieties used in Australia from the early 20^th^ century to the present. Co290 and NCo310 are foreign varieties from India and South Africa respectively and Comus is a noble cane (*Saccharum officinarum)*. The remaining twenty seven varieties are cultivars bred in Australia using local and foreign germplasm. DNA extraction was done on freeze dried leaves with the Qiagen DNeasy plant mini kit. DNA concentrations of the samples were determined using Qubit dsDNA HS assay and each sample was then diluted to a concentration of 20 ρg.µl^−1^. Final concentrations were checked, in sextuplicate, using the Qubit dsDNA HS assay. The ddPCR reactions were performed, in quadruplicate, according to manufacturer’s instructions, on a Biorad Qx200 system equipped with an automatic droplet generator. Each reaction passed the ddPCR quality control with a number of droplets above 10000 (Supplementary S3). Briefly, each 25 µL reaction well contained 5 µL of DNA, 12.5 µl of ddPCR Supermix for Probes (without dUTP), 1.25 µL of ITS2-pb2 custom TaqMan assay that contains primers and probe, 0.1 µL of the restriction enzyme *Alu*I and 6.2 µL of water. Plates were incubated at 37 °C for 30 min before the start of the automatic droplet generation. ddPCR results were converted to ITS copy number per ρg of DNA according to the following formula:

$$\begin{array}{c}{\rm{ITS}}\,{\rm{copy}}\,{\rm{number}}=[(\mathrm{copy}\,{\rm{nb}}\,{\mu L}^{-1}\times {\rm{reaction}}\,{\rm{volume}}/{\rm{volume}}\,{\rm{of}}\,{\rm{DNA}}\,{\rm{in}}\,\mathrm{reaction})\\ \,\,\,\,\,\,\,\,\,\,\,\,/{\rm{sample}}\,{\rm{concentration}}\end{array}$$

### Assay calibration based on root weight

In order to capture variation in this relationship due to diversity in root system morphology, six sugarcane varieties were selected. These six varieties have been previously identified for their contrasted root system morphology, especially in terms of average root diameter (Pierre *et al., unpublished*).

Six sugarcane varieties were selected (KQ228, MQ239, Q151, Q208, Q242 and SRA1). Three replicate plants from each variety were grown from section of stalk containing a single nodal bud (sett) in tall PVC pots (22.5 cm × 100 cm) for about 110 days (30 °C/16 h day, 24 °C/8 h night) in UC soil mix (50% sand, 50% peat) in non-limiting water and nutrient conditions. At this stage, roots had reached the bottom of the pot. At harvesting, root systems were washed thoroughly, split into two parts (0–50 cm and 50–100 cm from the crown) and then dried for at least 24 h at 45 °C in a large desiccating oven.

Samples were coarsely blended and carefully weighed to obtain standard ranges from 60 mg to 600 mg with 60 mg increments as well as two additional points at 900 and 1200 mg. A total of 144 calibration data points were generated from these samples. Samples were then sent to the SARDI commercial DNA extraction service (Adelaide, South Australia) where, prior to extraction, each sample was mixed with 200 g of sand. The DNA extractions and sugarcane root DNA quantification using the TaqMan assay were also conducted at SARDI as described.

### Rate of decay of root DNA in soil

Sixty-two soil samples of 400 g were prepared from moist topsoil (air-dried soil, water content 21%) collected on a bare patch 5 m from a sugarcane field trial located in Gatton, QLD, Australia (S27°32′23.7, E152°20′21.0). Half of the soil samples were dried for 48 h at 25 °C in a dehydrating oven while the other half were maintained under a humid atmosphere to preserve soil properties until the start of the experiment. Moist and dry soil samples were then spiked with 200 mg of lyophilized root segments (5 cm long) coming from a mix of the six sugarcane varieties used for the assay calibration and grown aeroponically. Two additional un-spiked moist soil samples were prepared for use as a negative control. Samples were incubated at 25 °C in a growth cabinet for 0, 1, 3, 5, 7 days. Six replicates were prepared for each time point/treatment combination. After incubation the samples were transferred to a dehydrating oven at 45 °C and were kept there until being send to SARDI for DNA extraction and analysis, as described.

### Assay validation

The assay was validated on field samples to assess: (i) the effect of controlled release fertiliser compared to urea on root systems; and (ii) the root system viability after harvest.

### Effect of nitrogen regime on root mass in the soil

Soil cores were collected on the 12^th^ and 13^th^ of June 2017 from a field trial managed by Herbert Cane Productivity Services Ltd (HCPSL) and located near Ingham, QLD, Australia (S18°36′11.0, E146°03′01.0). This was a second ratoon trial (harvested on the 29/10/2016) of the sugarcane variety Q208 that was established to test the effect of controlled released fertiliser compared to conventional fertiliser on sugarcane performance.

Soil core samples were collected for three different experimental treatments: 140 kg of N/ha as urea, 140 kg of N/ha as 75% polymer-coated urea and 25% urea, and 0 N kg/ha. Each experimental treatment was replicated four times in the field. Soil sampling was done using a Dig Stick Soil Probe (Ø15mm × 1100 mm) hammered down to the depth of 45 cm. After retrieval, soil cores were divided into 15 cm segments. Each sample was formed from a composite of 12 cores, from the same depth, for a total maximum weight of 500 g. A total of 72 composite samples was collected. Samples were kept on ice in the field and then transferred to an oven to be dehydrated at 45 °C for at least 24 h before being sent to SARDI for DNA extraction and analysis, as described. The results expressed as the number of copies of the target DNA sequence per gram of soil were converted to root dry mass per volume of soil using the Q208-50 cm linear regression obtained from the calibration experiment and an average soil bulk density of 1.5 g cm^−3^.

### Root turnover after harvest

Soil cores were collected from an irrigated second ratoon field trial managed by CSIRO located near Gatton, QLD, Australia (S27°32′23.7, E152°20′21.0). Samples were collected from the following sugarcane varieties: MQ239, Q151, SRA1 and Q240. Half of the Q240 plants were not harvested until the last sampling date and were therefore used as a positive uncut control. Two cores were collected per plant on four different plants per variety and the same plants were used for the duration of the experiment. Soil cores were collected with a Dig Stick Soil Probe (Ø15mm × 1100 mm) hammered down to the depth of 60 cm. Each soil core was then divided into 15 cm segments. Each composite sample was made up of eight soil core segments. Cores were collected on the day of harvest (T0, 24/08/17), one week after harvest (T1, 31/08/17) and two weeks after harvest (T2, 08/09/17). After collection, samples were kept on ice until transfer to a dehydrating oven at 45 °C for at least 24 h. Samples were then sent to SARDI for DNA extraction and analysis, as described.

### DNA extraction and sugarcane root DNA quantification

DNA was extracted from root and soil samples, using the commercial DNA extraction service operated by SARDI, Adelaide^12^. The efficiency and consistency of SARDI’s method to extract DNA from soil has been confirmed in comparison to commercial methods^15^. Prior to DNA extraction, a standard amount of internal control was added to each sample to assess both DNA extraction efficiency and PCR inhibition (Supplementary S2). Sugarcane root and internal control DNA were quantified by qPCR performed on QuantStudio7 real-time PCR system (Applied Biosystems, Foster City, CA, USA), using the PCR conditions described above. Each PCR plate included no-template controls as well as calibration standards to calculate the amount of sugarcane root DNA per sample; results were reported as ‘Number of copies of the target DNA sequence per gram of sample’. None of the 194 soil samples failed the internal control quality check and only one of the 144 assay calibration samples (root plus sand) showed a slight PCR inhibition, confirming the robustness and consistency of the DNA extraction protocol.

### Statistical analysis

All the statistical analyses (linear regression, coefficient of determination, analysis of variance) and graphics were conducted in R^27^ using the tidyverse package^28^.

## Electronic supplementary material

Supplementary material

## Data Availability

The datasets generated and analysed for this work are available from the corresponding author on request.
