# Supplementary material for: DNA based diagnostic for the quantification of sugarcane root DNA in the field
Source: Sci Rep. 2018 Nov 13;8:16720. doi: 10.1038/s41598-018-34844-3 (PMC6233164; doi:10.1038/s41598-018-34844-3)
Supplement: Supplementary file 1 — Supplementary material [file 41598_2018_34844_MOESM1_ESM.docx]

**TITLE**

DNA based diagnostic for the quantification of sugarcane root DNA in the field.

**AUTHORS**

J.S. Pierre^1*^, D. Giblot-Ducray^2^, A.C. McKay^2^, D.M. Hartley^3^*,* J.M Perroux^1^, A.L. Rae^1^.

1. CSIRO Agriculture and Food, 306 Carmody Road, St Lucia, QLD 4067, Australia.
2. Molecular Diagnostics Centre, South Australian Research and Development Institute, 2b Hartley Grove, Urrbrae, SA 5064, Australia.
3. CSIRO National Research Collections Australia, Canberra, ACT 2601, Australia

**CORRESPONDING AUTHOR**

[johann.pierre@csiro.au](mailto:johann.pierre@csiro.au) , +61732142267.

**Supplementary material**

**Supplementary S1.** ITS1 and ITS2 consensus sequences obtained from the sequencing of 18 sugarcane variety. N in red represents SNP. ITS. 1 ITS1 was too rich in SNPs and therefore not suitable for the design of a robust and specific TaqMan assay; ITS2 was preferred instead.

**Supplementary S2.** Summary table of the Ct value differences between the internal and inhibition controls used to control for the efficiency and consistency of the DNA extraction and amplification.

| **Experiment** | **Number of samples** | **Ct value difference to the inhibition control**  **mean ± sd** |
| --- | --- | --- |
| Calibration_plate1 | 84 | 0.05 ±0.11 |
| Calibration_plate2 | 60 | 0.07 ±0.05 |
| Root DNA decay | 62 | 0.23 ±0.08 |
| Nitrogen fertilizer | 72 | 0.12 ±0.07 |
| Root turnover | 60 | 0.16 ±0.09 |

**Supplementary S3.** Summary table of the number of positive and negative droplets used in the ddPCR experiments to calculate ITS copy number for the 31 sugarcane cultivars.

| **Cultivar** | **Mean positive droplets ±sd** | **Mean negative droplets ±sd** |
| --- | --- | --- |
| Co290 | 15248 ±816 | 3606 ±180 |
| Comus | 16296 ±958 | 1721 ±132 |
| EMPIRE | 17656 ±322 | 1174 ±52 |
| KQ228 | 17650 ±758 | 1026 ±41 |
| MQ239 | 16124 ±918 | 2191 ±125 |
| NCO310 | 16232 ±1175 | 398 ±31 |
| Pindar | 14500 ±449 | 3487 ±161 |
| POJ2878 | 15770 ±352 | 1943 ±28 |
| Q113 | 17807 ±733 | 367 ±35 |
| Q117 | 15867 ±976 | 3085 ±128 |
| Q124 | 16873 ±597 | 165 ±22 |
| Q138 | 16353 ±1037 | 790 ±66 |
| Q151 | 17175 ±193 | 1551 ±86 |
| Q167 | 16637 ±525 | 2209 ±82 |
| Q190 | 16100 ±825 | 1375 ±131 |
| Q200 | 16589 ±493 | 1825 ±141 |
| Q208 | 16375 ±1654 | 221 ±42 |
| Q231 | 15538 ±1686 | 1215 ±100 |
| Q232 | 16639 ±918 | 1273 ±66 |
| Q234 | 18470 ±1133 | 742 ±61 |
| Q242 | 16152 ±408 | 2118 ±77 |
| Q249 | 16166 ±582 | 2812 ±157 |
| Q252 | 17614 ±265 | 2116 ±79 |
| Q256 | 15378 ±1105 | 1436 ±106 |
| Q77 | 15259 ±1406 | 1951 ±160 |
| Q96 | 16415 ±504 | 2535 ±88 |
| QBYCO5-20853 | 17865 ±978 | 1306 ±96 |
| QC91-580 | 16075 ±1105 | 1657 ±119 |
| QN04-121 | 16654 ±1954 | 809 ±126 |
| QN04-668 | 17492 ±904 | 933 ±35 |
| SRA1 | 18084 ±762 | 813 ±61 |
